# Initial validation of the Multidimensional Adolescent Functioning Scale (MAFS) in Spanish-speaking students from Chilean secondary schools

**DOI:** 10.1186/s12955-023-02163-5

**Published:** 2023-07-24

**Authors:** Daniel Núñez, César Villacura-Herrera, Jorge Gaete, Daniela Meza, Javiera Andaur, Johanna T.W. Wigman, Jo Robinson

**Affiliations:** 1 Centro de Investigación en Ciencias Cognitivas, Faculty of Psychology, Universidad de Talca, Talca, Chile; 2 Millennium Science Initiative Program, ANID, Millennium Nucleus to Improve the Mental Health of Adolescents and Youths, Santiago, Chile; 3 Facultad de Educación, Universidad de Los Andes, Las Condes, Chile; 4 Department of Psychiatry, Interdisciplinary Centre for Psychopathology and Emotion regulation (ICPE), University Medical Center Groningen, Groningen, the Netherlands; 5 Orygen, Parkville, VIC 3052 Australia; 6 Centre for Youth Mental Health, University of Melbourne, Parkville, VIC 3010 Australia

**Keywords:** Multidimensional adolescent functioning scale, Psychosocial functioning, Adolescents

## Abstract

**Introduction:**

Psychosocial functioning is closely associated with psychopathology and wellbeing in different populations, particularly adolescents. Despite its relevance, measures assessing psychosocial functioning in healthy adolescents are scant as most focus on adults or clinical populations. We evaluated the psychometric properties of the Multidimensional Adolescent Functioning Scale (MAFS), a self-report questionnaire created to assess three dimensions of psychosocial functioning (‘general functioning’, ‘family-related functioning’, and ‘peer-related functioning’) in adolescents from the general population.

**Methods:**

After translation and cultural adaptation, we administered the Spanish MAFS to 619 adolescents aged 14 to 19. We assessed the factor structure, internal consistency, and associations with depressive symptoms, suicidal ideation, cognitive-behavioral skills, cognitive reappraisal (CR), and expressive suppression (ES). We additionally tested for measurement invariance based on biological sex.

**Results:**

The original three-factor structure showed the best fit. Internal consistency was good for the total scale (ω = 0.874; α = 0.869; GLB = 0.939, *r*_M_=0.216) and for all subscales (ω = 0.806-0.839; α = 0.769 to 0.812; GLB = 0.861-0.873). Correlations between all three MAFS subscales were significant, ranging between 0.291 and 0.554. All MAFS subscales correlated positively and significantly with cognitive-behavioral skills and adaptive regulatory strategies and negatively with depressive symptoms and suicidal ideation.

**Conclusion:**

The Spanish MAFS translation is a valid and reliable self-report measure to assess three domains of psychosocial functioning in adolescents aged 14–19 from the general population.

**Supplementary Information:**

The online version contains supplementary material available at 10.1186/s12955-023-02163-5.

## Introduction

Psychosocial functioning is associated with wellbeing and mental difficulties [1]. Despite the lack of consensus about its definition [2], it is generally accepted that it encompasses complex interactions including social cognitions, skills, and competencies, along with social interactions and behaviors [3] as well as feelings and thoughts about social situations [4]. Adaptive social functioning has been related, among others, to healthier lifestyles [5, 6], school engagement [7], and good interpersonal relationships [8]. By contrast, poor psychosocial functioning has been associated with school refusal [9], loneliness [10], emotion dysregulation [11], social anxiety and depression [12, 13], psychotic experiences [14], psychotic disorders [15–17], as well as with suicidal risk [18]. Both psychosocial functioning and mental health problems are particularly relevant during adolescence and are regarded as potential predictors for future adverse outcomes in adulthood [9, 19]. Regardless of its importance, there is a scarcity of multidimensional instruments assessing psychosocial functioning in adolescents beyond and independently from psychopathology and functional impairments [20].

Most of the available measures of psychosocial functioning are adult-focused tools that do not consider adolescents’ specific characteristics [21]. Second, the instruments usually evaluate both psychopathological symptoms and functioning, focusing on different dimensions of functional impairments. Third, psychosocial functioning has mostly been described using objective or observed-based indices instead of self-report tools [2]. Fourth, most tools measuring psychosocial functioning in children and adolescents have been designed for clinical populations and usually require parallel ratings by adults (parents or teachers). This approach is not always appropriate for adolescents, who are frequently reluctant to disclose their inner experiences, particularly those associated with critical domains such as peer relationships [21]. Thus, despite their multidimensional nature and easy use, they cannot accurately capture subtle changes in psychosocial functioning occurring in adolescents from the general population [20], which is encouraged nowadays [2].

Prior research has suggested that these limitations can be partially overcome by measuring functioning independently of psychopathology, as done by the Multidimensional Adolescent Functioning Scale (MAFS). This is a self-report questionnaire designed for general populations and populations at-risk for psychopathology. The MAFS assesses three domains: general functioning (GF), family functioning (FF), and peer functioning (PF). The original version was validated in adolescents from Australia [21], and its psychometric properties have recently been examined in adolescents from the Netherlands [20] and Iran [22], demonstrating good validity and reliability indices overall (α = 0.67-0.88; GLB = 0.75-0.86). Compared to other instruments, the MAFS offers some advantages: (1) it is a brief questionnaire and thus its burden is limited; (2) it was explicitly designed to measure adolescent functioning; (3) it provides multidimensional information on three aspects of psychosocial functioning relevant in this period; (4) it takes good psychosocial functioning as its reference point. These characteristics could increase the capability to accurately capture slight variations in functioning levels in non-clinical settings [20], following recent literature encouraging both conceptualizing and measuring mental health beyond psychiatric symptoms [19, 23].

To date, no previous studies examining the validity and reliability of the MAFS in Spanish-speaking countries have been conducted. Considering this gap and given both the suitability of self-report questionnaires to assess psychosocial constructs in adolescents [24] and the well-established need to validate specific instruments to evaluate adaptive social functioning in different cultural contexts [25], the present study sought to examine psychometric properties of the Spanish translation of the MAFS in a sample of adolescents in Chile.

### Current study aims

The aims of the present study were: (i) to evaluate competing models of the latent structure of the MAFS, using confirmatory factor analysis; (ii) to explore the reliability of the resulting sub-scales having the best fit; (iii) to examine associations between psychosocial functioning and several other domains such as psychopathology (depression and suicidal ideation), cognitive-behavioral skills, and emotion regulation strategies to assess external validity. We hypothesized that the proposed three-factor structure would show the best fit and that the MAFS would show good reliability indices. Moreover, we expected to find positive associations with higher cognitive-behavioral skills and adaptive emotion regulation, and negative associations with mental health symptoms and maladaptive emotion regulation. Last, we tested for psychometric equivalence of the psychosocial functioning construct across biological sex groups (male/female), aiming to confirm the utility of the MAFS as an assessment tool for both males and females.

## Method

### Participants

Using a convenience sampling method, six-hundred and thirty-four participants aged 14–21 years (M = 16.000; SD = 1.420) were recruited from ten secondary public schools from Chile as part of a larger research project focused on secondary students. Due to the MAFS being constructed for assessing functionality in adolescents, we considered the 10 to 19 years old age range established by the World Health Organization [26]. Based on these criteria, a total of fifteen participants aged over 19 years (2.37%) were excluded from the study. Thus, the final sample included a total of 619 adolescents aged 14–19. Participants did not receive any financial or academic incentive for participating in the study. Table 1 presents a summary of the characteristics of the final sample.

**Table 1:** Sample characteristics

		*n*(%) or M(SD)			Range
Biological sex					
	Male	302	(48.79%)		
	Female	317	(51.21%)		
Psychiatric treatment					
	No	339	(54.77%)		
	Past	203	(32.79%)		
	Current	77	(12.44%)		
Nationality					
	Chilean	600	(96.93%)		
	Foreign	19	(3.07%)		
Age (years)					
	Male	15.95	(1.39)		14–19
	Female	15.93	(1.30)		14–19
	Total	15.94	(1.34)		14–19
MAFS					
	GF	28.89	(5.44)		13–39
	FF	18.80	(3.04)		9–24
	PF	12.96	(3.41)		6–20
ERQ-CA					
	CR	20.66	(3.13)		6–30
	ES	13.27	(3.36)		4–20
CBTSQ					
	CgR	19.97	(5.96)		7–35
	BA	19.46	(6.09)		7–35
SIQ-JR					
	AI	9.57	(6.32)		6–42
	GI	15.75	(9.05)		6–42
	IP	6.39	(4.83)		3–21
PHQ-9					
	Total score	10.97	(6.44)		0–27

MAFS = Multidimensional Adolescent Functioning Scale; GF = General functioning; FF = Family-related functioning; PF = Peer-related functioning; ERQ-CA = Emotion Regulation Questionnaire for Children and Adolescents; CR = Cognitive reappraisal; ES = Expressive suppression; CBTSQ = Cognitive Behavioral Therapy Skills Questionnaire; CgRs = Cognitive restructuring; BA = Behavioral activation; SIQ-JR = Suicide Ideation Questionnaire Junior; AI = Active ideation; GI = General ideation; IP = Interpersonal problems; PHQ-9 = Patient health questionnaire

### Procedure

Ten public schools were invited to participate in the study. After meetings with their administration teams, where they received information on the research and its objectives, all schools agreed to participate. Upon obtaining each school’s written approval, students and their caregivers were contacted and informed at parents’ meetings of the characteristics of the study. Voluntary participation and confidentiality, along with information on action protocols in case of detecting potential risk or need for psychological assessment, were explicitly stated. Once written informed consent was obtained from both the students and their caregivers, the participants completed an online survey. Ethical approval was obtained from the Scientific Ethics Committee of the Universidad de Talca.

### Measures

#### Psychosocial functioning

We used the Multidimensional Adolescent Functioning Scale (MAFS) [21]. This 23-item self-report instrument assesses three categories of adolescent functioning: general functioning (GF), family-related functioning (FF), and peer-related functioning (PF). Responses are ranged from 1 (not at all or rarely) to 4 (Always or almost always). Each subscale score is determined by adding the item scores from each respective scale. The GF subscale comprises ten items with a total scoring range of 0–40, the FF subscale includes seven items with a total scoring range of 0–28, and the PF subscale consists of six items with a total scoring range of 0–24. Higher scores indicate better functioning in each area. According to prior studies, its internal consistency has been good, with scores ranging from 0.67 to 0.88 in samples from the general population [20–22].

The English version of the MAFS was translated and adapted for Spanish speakers, following recommendations by Muñiz et al. [27]. Three Spanish-speaking psychologists (PhD) independently translated all items. No additional changes were made because of the high interrater agreement obtained during the first back translation. The agreement was reached by consensus, based on a panel discussion.

#### Emotion regulation

We used the Emotion Regulation Questionnaire for Children and Adolescents (ERQ-CA) [28], Spanish version [29, 30]. It is a 10-item scale with Likert responses ranging from 1 (totally disagree) to 5 (absolutely agree) that assesses two major dimensions: cognitive reappraisal (CR, six items) and expressive suppression (ES, five items) (ES, four items). A higher score indicates greater use of each ER strategy. Previous research has found that its subscales have strong internal consistency (Cronbach’s alpha ranging from.71 to.83) [28, 31]. Internal consistency in our sample was satisfactory for both subscales (CR: ω = 0.762, α = 0.761, *r*_M_=0.348; ES: ω = 0.718, α = 0.713; *r*_M_=0.383).

#### Cognitive-behavioral therapy skills

We used the Cognitive-Behavioral Therapy Skills Questionnaire (CBTS) [32]. This 16-item scale measures two skills: cognitive restructuring (CgRs) and behavioral activation (BA) (i.e., changes in avoidance/ behavioral control and changes in cognitive style). Respondents rank each item on a 5-point Likert scale from 1 (I don’t do this) to 5 (I always do this). The minimum score is 15, and the maximum score is 80. A higher score means a higher presence of cognitive-behavioral skills. The authors reported the measure to have shown good fit and internal consistency indices, with Cronbach’s alpha values of 0.88 for the CgRs subscale and 0.85 for the BA subscale. In our sample, internal consistency indices were good for the CBTSQ (ω = 0.843, α = 0.841, *r*_M_=0.276) and for each factor (CgRs: ω = 0.806, α = 0.805, *r*_M_=0.374; BA: ω = 0.819, α = 0.818, *r*_M_=0.391).

#### Depressive symptoms

We used the Patient Health Questionnaire-9 (PHQ-9) [33], a nine-item self-report questionnaire with response options ranging from 0 (not at all) to 3 (nearly every day). The maximum score range is 0 to 27. Scores between 0 and 4 indicate the absence of depressive symptoms, 5–9 mild to moderate symptoms, 10–14 moderate depressive symptoms, 15–19 fairly severe symptoms, and 20–27 severe symptoms [33]. Borghero et al. observed good internal consistency (α = 0.78), sensitivity (86.2%), and specificity (82.9%) in a sample of Chilean adolescents [34]. Reliability indices in our sample were good for the total score (ω = 0.892, α = 0.889, *r*_M_=0.473).

#### Suicidal ideation

We utilized the Suicidal Ideation Questionnaire for Children (SIQ-JR), a 15-item self-report questionnaire designed to detect suicidal thoughts among adolescents [35]. Values are scored from 0 to 6, with higher scores indicating more severe suicidal ideation. The measure has a maximum score of 90, with a threshold of 31 indicating a clinically significant level of suicidal ideation. Previous research has shown high levels of internal consistency (α = 0.978) and construct and criterion validity [36, 37]. In our sample, the internal consistency indices were good to very good for both the entire scale (ω = 0.955, α = 0.951, *r*_M_=0.577) and each subscale (AI: ω = 0.921, α = 0.905, *r*_M_=0.608; GI: ω = 0.896, α = 0.894, *r*_M_=0.591; and IP: ω = 0.909, α = 0.909, *r*_M_=0.772).

### Statistical analysis

Kaiser-Meyer-Olkin (KMO) and Bartlett’s sphericity tests were conducted to ensure the suitability of the sample for factor analysis (expected: KMO ≥ 0.800; Bartlett’s *p* < .05). To test the internal structure of the scale, we performed confirmatory factor analysis (CFA) using a robust maximum likelihood (RML) estimator. A three-factor model CFA was performed using the original structure described by Wardenaar et al. [21]. We also tested a single-factor model to assess if a simpler structure would fit the data better. Additionally, based on the results obtained by Mayle et al. [20], we tested a bifactor model with a common latent factor (CLF). The models were compared based on well-known fit indices such as Chi-square divided by degrees of freedom ratio (χ^2^/df), the root mean square error of approximation (RMSEA), the standardized root mean square residual (SRMR), the comparative fit index (CFI), the Tucker-Lewis index (TLI) and the goodness of fit index (GFI). Comparisons were performed based on their closeness to established cut-off values (χ^2^/df < 5; RMSEA < 0.08; SRMR < 0.08; CFI ≥ 0.90; TLI ≥ 0.90; GFI ≥ 0.95) [38]. We additionally compared model parsimony using indices such as the Akaike information criterion (AIC) and the Bayesian information criterion (BIC), where lower values in both indices could provide evidence for better fit and model parsimony.

Internal consistency and reliability of all measures and their subscales were calculated through McDonalds’ omega (ω), Cronbach’s alpha (α), the greatest lower bound (GLB), and the average inter-item correlation (*r*_M_). Interpretation of ω and α coefficients was based on commonly accepted cut-off criteria, where values above 0.70 are deemed acceptable, above 0.80 are considered good, and above 0.90 are viewed as excellent for the internal consistency of the measure [39, 40]. The greatest lower bound (GLB) is often considered a more accurate estimate of reliability, and although no universally accepted cut-off exists, it is generally agreed that values closer to 1 indicate excellent reliability [41, 42]. For the average inter-item correlation (rM), acceptable values generally range from 0.15 to 0.50, representing a balance between item redundancy and internal consistency [43].

Next, we tested for psychometric equivalence of psychosocial functioning measurement between males and females through assessment of measurement invariance. For this, we conducted sequential tests to examine for invariance at different levels [44, 45]. The first level, known as configural, examines whether the same items measure the same constructs for both groups. If configural invariance is established, the next step is to test for metric invariance, which examines if factor loadings are equivalent between the two groups. Likewise, if metric invariance is established, scalar invariance is tested, which examines for equivalence of item intercepts between the groups. Last, if all other previous levels are established strict invariance is examined, which tests for equivalence of item residuals across groups. These levels test for equivalence of free and fixed factor loadings, equivalence of item loadings, equivalence of item intercepts and equivalence of item residuals across groups [44–47].

To establish measurement invariance, it is expected to observe low differences in fit indices such as CFI (ΔCFI ≤ 0.01) between models such as and non-significant changes in χ^2^ (pΔχ²>0.05) and RMSEA, tested by computing the Probability of close fit at every level (*p*Close > 0.05). Also, due to their robustness when working with more complex models, we calculated Gamma Hat (γ̃) and McDonald’s noncentrality index (NCI), which are independent from other fit indices, while also being robust to sample size and intricate model structures [48, 49]. Cut-off criteria is based on the critical values proposed by Cheung & Rensvold [49].

When establishing a certain level of invariance was not possible due to significant differences between models, we proceeded to examine for partial invariance at that particular stage. Partial invariance assumes that some items may not behave equally across groups, thus the items’ equality constraints are relaxed sequentially in order to detect those least invariant [45, 50]. Partial invariance is achieved when, for each factor, a minimum of two items demonstrate consistent measurement properties across different groups [45]. This ensures that valid comparisons can still be made while acknowledging the inherent variability associated with each group [47, 50, 51].

Through Spearman’s correlation coefficient, we assessed associations between the MAFS subscales, CBTSQ subscales, the total score of the PHQ-9 and all three SIQ-JR subscales. We expected that scores in the MAFS would correlate positively with the ERQ-CA’s CR and the CBTSQ’s CgR and BA subscales, while correlating negatively with the ERQ-CA’s ES scale, all SIQ-JR subscales and the PHQ-9 total score.

Statistical analyses were performed using JASP v0.16.2 [52] and IBM Amos Graphics v26. For JASP’s output to be consistent with Amos’, EQS emulation was selected.

## Results

### Internal structure

KMO and Bartlett’s sphericity tests yielded satisfactory results, allowing us to conclude that our data is suitable for factor analysis (KMO = 0.885; χ^2^_Bartlett_ = 4966.876; *p* = .000).

**Fig. 1:**
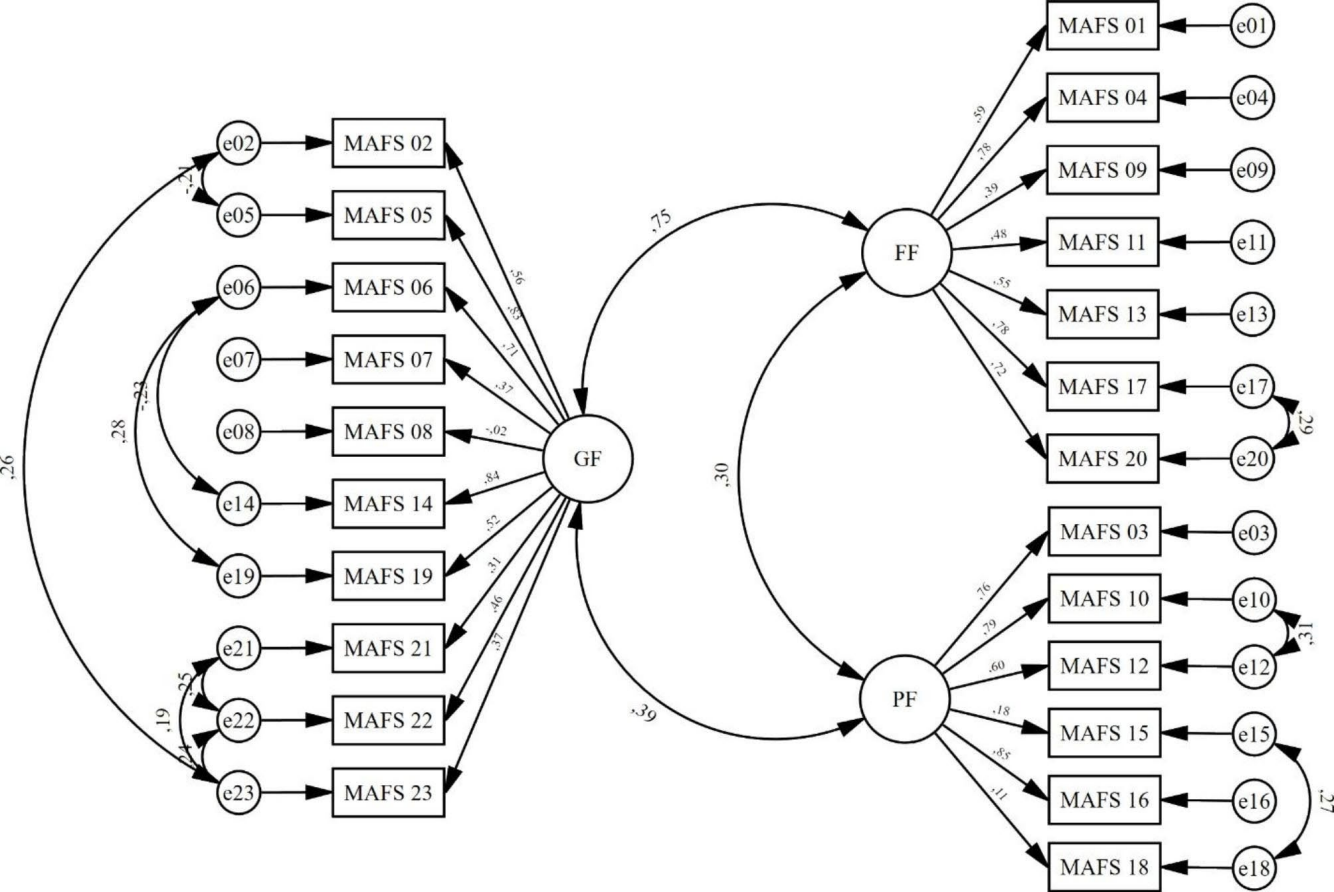
Path diagram for the final three-factor model of the MAFS showing standardized factor loadings and regression coefficients

CFA results showed that the original three-factor structure demonstrated relatively good fit indices, whereas the single-factor structure showed poor fit and lower parsimony overall (Table 2). Thus, the original three-factor model was chosen as the best fitting model. However, indices such as CFI and TLI remained suboptimal compared to established cut-off values. Thus, we examined modification indices (MI) for each parameter. Examination of MI revealed that model fit would improve by allowing residuals of paired items 2–5, 2–23, 6–14, 6–19, 21–22, 21–23, 22–23, 17–20, 10–12, 15–18 to covariate (MI > 18.900). Bivariate correlations between these items were generally moderate (*r* = .285 to 0.540), suggesting they are heterogeneous enough to be considered independent [53]. As a side note, correlations between items 10 (“*I feel close to my friends*”) and 12 (“*I spend quite a lot of time with my friends*”), and items 17 (“*My family is supportive of me when I need it*”) and 20 (“*My parents are encouraging*”) were noticeably higher (*r* > .60). Figure 1 presents the path diagram for the final three-factor model after MI examination.

**Table 2:** Confirmatory factor analysis (CFA)

	Absolute fit						Relative fit				Parsimony	
	χ²	df	χ²/df	RMSEA	SRMR		CFI	TLI	GFI		AIC	BIC
Single-factor	1972.939	230	8.578	0.111	0.092		0.631	0.594	0.745		32519.305	32722.998
3-factor (original)	809.113	227	3.564	0.064	0.058		0.877	0.863	0.890		31359.596	31576.573
3-factor (MI)	483.073	217	2.226	0.045	0.051		0.944	0.934	0.936		31053.028	31314.286
Bifactor (CLF)	584.740	207	2.825	0.054	0.045		0.920	0.902	0.919		31174.859	31480.398

RMSEA = Root mean square error of approximation; SRMR = Standardized root mean square residual; CFI = Comparative fit index; TLI = Tucker-Lewis index; GFI = Goodness of fit index; AIC = Akaike information criterion; BIC = Bayesian information criterion; MI = After modification indices examination; CLF = Common latent factor

### Internal consistency

We found good values in terms of internal consistency for the full MAFS scale (ω = 0.874, α = 0.869, GLB = 0.939, *r*_M_=0.216) and for all three subscales (GF: ω = 0.805, α = 0.788, GLB = 0.865, *r*_M_=0.263; FF: ω = 0.833, α = 0.812, GLB = 0.861, *r*_M_=0.375; PF: ω = 0.839, α = 0.769, GLB = 0.873, *r*_M_=0.302).

### Measurement invariance

Results of measurement invariance tests at configural to scalar levels on the MAFS are detailed in Table 3. Invariance was established at the configural level. However, we found non-invariance at a metric level after constraining all factor loadings. Thus, a partial metric invariance model was tested where the factor loading of item 13 (“*members of my family are disappointed in me”*) was left unconstrained due to showing the largest variability between males and females. With this, partial invariance could be established. Next, we tested for scalar invariance by adding equality constraints to all intercepts but were unable to establish invariance at this level. Multiple partial scalar models were also tested, but the results were unsatisfactory. Thus, scalar biological sex invariance could not be confirmed in our sample.

**Table 3:** Measurement invariance tests

	Model fit							Invariance tests							
	χ²	df	CFI	NCI	γ̃	RMSEA		Δχ²	Δdf	*p*_Δχ²_	ΔCFI	ΔNCI	Δγ̃	ΔRMSEA	*p*Close
Configural	727.549	434	0.937	0.788	0.960	0.041		—	—	—	—	—	—	—	—
Metric	760.933	454	0.934	0.780	0.956	0.047		33.384	20	0.031	− 0.003	− 0.008	− 0.004	0.006	1.000
Partial metric	751.177	453	0.936	0.786	0.957	0.033		23.628	19	0.211	− 0.001	− 0.002	− 0.003	− 0.008	1.000
Scalar	906.899	474	0.907	0.705	0.940	0.054		155.722	21	0.000	− 0.081	− 0.017	− 0.017	0.021	1.000

CFI = Comparative fit index; NCI = Non-centrality index; γ̃=Gamma hat; RMSEA = Root mean square error of approximation; Δ = Difference with previous level; *p*Close = Probability of close fit

Mean comparisons between males and females for the MAFS scores showed statistically significant results on the GF (M_dif_=1.793; *t* = 4.145; *p* = .000; *d* = 0.333), FF (M_dif_=1.361; *t* = 5.703; *p* = .000; *d* = 0.459) and PF subscales (M_dif_=1.230; *t* = 4.544; *p* = .000; *d* = 0.365). In all cases, male participants showed higher functioning levels than females. Comparisons for the rest of the measures are detailed in Supplementary Table S1.

We also found positive and significant correlations between all three MAFS subscales and adaptive emotion regulation strategies and cognitive-behavioral skills (*r* = .080 to 0.562; *p* < .05), while finding negative correlations with maladaptive emotion regulation strategies, suicide ideation, and depressive symptoms (*r*=-.107 to − 0.641; *p* < .05). Results are detailed in Supplementary Table S2.

## Discussion

This is the first study providing evidence on the factor structure and reliability of the Spanish translation of the MAFS, a self-report measure assessing three dimensions of psychosocial functioning in Spanish-speaking adolescents. Our results support the original three-factor structure previously observed in adolescents in Australia [21] and Iran [22]. Internal consistency coefficients were found to be good for the full scale and each subscale. Moreover, we found significant and theoretically meaningful associations between psychosocial functioning and psychopathology, such as depressive symptoms and suicide ideation, along with other transdiagnostic processes, such as cognitive-behavioral skills and emotion regulation strategies. These results align with prior studies showing clear links between psychosocial functioning levels and positive [6] and psychopathology-related [54] outcomes in adolescents.

Only three studies have previously assessed the psychometric properties of the MAFS. Our findings in terms of its structure are consistent with the three-factor model previously found across populations aged 15–18 [21] and 15–17 [22] However, the study by Mayle et al. [20] with adolescents ranging from 12 to 17 years old found a better fit testing a bifactor model with four uncorrelated factors (a general factor loading on all items (MAFS-general) and three group factors, loading respectively on GF, FF, and PF). In line with this, we tested the same bifactor model and observed that it fit our data well. However, after the MI examination, the original three-factor structure was demonstrated to be the best fitting overall. Then, similar to the other three MAFS questionnaires, the Spanish version also showed a multidimensional structure with separate and correlated domains of general, family and peer-related functioning. This multidimensional nature is particularly crucial when assessing functioning in adolescents from the general population, for it allows for accurate measurement of specific areas that can be potentially different in this stage, such as with family and peers [55]. Evidence shows that perceived social support undergoes significant changes during adolescence, with perceived family support decreasing and perceived peer support increasing [56]. Critical to the understanding of an adolescent’s psychosocial functioning is the degree to which they feel supported by others, as it has the potential to significantly impact attitudes toward various social groups and, as a result, influence how the adolescents interact with those groups [57].

Regarding reliability indices of the measure, our results were highly similar to prior studies showing good internal consistency for the full scale and all subscales of the MAFS. Our findings on differences between males and females indicate that males showed higher psychosocial functioning levels than females in all three dimensions: GF, FF, and PF. This contrasts with Mayle et al. [20], who found higher scores in females for the peer functioning subscale. Moreover, our finding also differs from Price et al. [25], who reported higher levels of social functioning (general, peer relationships, and home duties/self-care) in female adolescents aged 12 to 14 years when assessed by the Social and Adaptive Functioning for Children and Adolescents Scale. Our results on biological sex differences show that males seem to have better levels of -at least perceived- psychosocial functioning compared to females. These discrepancies between the results of the two studies may be attributable to several factors, including cultural differences and item interpretation. Additionally, a reporting bias where males could be less inclined to share their vulnerabilities, cannot be ruled out. Since this is the first study examining measurement invariance of MAFS overall, further evidence is still required to determine whether the observed differences in the measurement of psychosocial functioning between males and females are attributable to sample characteristics, cultural factors, other developmental variables, or the way the items are described. In addition, because measurement invariance could only be confirmed at configural and partial metric levels, we cannot assume that the latent constructs of psychosocial functioning (GF, FF and PF) and its item residuals are fully equivalent across groups.

While no other studies exist examining this aspect of the MAFS, studies testing for measurement invariance in other social functioning questionnaires in adolescents are scant. Our results differ from Gonzálvez et al. [1] who found evidence for invariance between males and females in a sample of children aged 8–12 years who answered the Child and Adolescent Social Adaptive Functioning Scale (CASAFS) [58]. Our findings also partially fit with Karcher and Sass [59] who suggest that, from a practical perspective, the Measure of Adolescent Connectedness could be used for assessment purposes in male and female children, although the partial invariance revealed some group difference. Evidence for non-invariance across these groups might reflect some characteristics of our sample in terms of the significant differences observed for males and females in general. For instance, we found that males showed lower depressive symptoms and suicidal ideation levels, lower usage of emotional suppression and higher usage of cognitive reappraisal and behavioral activation strategies. This fits with recent research showing that general psychopathology correlates with social interaction quality in young people [54]. Further research assessing the interaction of psychopathological domains, regulatory processes and social functioning in adolescence through longitudinal approaches is needed.

Considering the well-established associations between psychosocial functioning and both positive outcomes (e.g., higher levels of wellbeing) as well as negative ones (e.g., higher levels of psychopathology), our study explored for the first-time relationships between the MAFS and depressive symptoms, emotion regulation strategies, and suicidal ideation. Our findings are consistent with research revealing negative associations with depression [16], expressive suppression [60], and suicidal ideation [13]. Moreover, we found that all subscales were positively associated with both adaptive emotion regulation strategies (cognitive reappraisal) and cognitive-behavioral skills, such as CgR and BA. This fits with prior research revealing that adolescents with higher social functioning presented higher adaptive regulatory strategies [61]. Our findings also align with Mayle et al. [20], who observed specific associations between cognitive reappraisal and happiness and the two most general factors underlying the MAFS (the common latent factor and GF) and between prosocial behaviors and peer and functioning. Our results suggest that the MAFS could provide valuable information on the associations between psychosocial functioning and a wide range of dimensions encompassing healthy and unhealthy related outcomes, beyond the associations previously observed between psychotic experiences in adolescents [62–64]. This fits with the general frame encouraging a social perspective on mental health care [65], emphasizing social interactions as core factors for understanding mental disorders [66]. Our results may help to include psychosocial functioning as valuable intervention outcomes and explore the mechanisms of how these interventions work in the future. It is worth mentioning that although our study captured a significant proportion of the adolescent years, not all ages that according to the WHO definition were included, especially the early years (10–14). However, this age range encompasses a substantial portion of the adolescent period, particularly the later years where individuals have likely experienced some or most of the physical, emotional, social and mental changes associated with adolescence [67–69].

### Limitations

Some limitations deserve to be mentioned. First, because of our cross-sectional design, we did not assess temporal stability and cannot establish causal relationships among variables. Second, we only recruited participants from public schools. Therefore, adolescents from higher socioeconomic backgrounds, usually attending private schools, could be underrepresented. Third, we did not include younger adolescents (10–13 years old) as participants from that age range were unavailable in our sample of secondary students. Specifically, our findings might be less applicable to adolescents under 14. Furthermore, in our sample, 13.23% of the adolescents were 18–19 years, and thus, older adolescents were underrepresented. This is relevant considering the age-dependent variability of behavioral and cognitive capacities underlying social functioning in adolescents and the dynamic nature of this construct during the life course [2].

## Conclusions

The present study demonstrates that the Spanish version of the MAFS is a reliable self-report measure of psychosocial functioning in adolescents aged 14–19 years. Adolescence is, in essence, a dynamic and multidimensional stage characterized by gradual psychosocial and functional changes [67, 70, 71]. Moreover, most mental illness often emerges during this period characterized by mood oscillations and transient periods of negative affects strongly associated with social functioning [72–74].

The meaningful associations with positive and negative outcomes suggest that this measure could provide helpful information in clinical and non-clinical settings, also being suitable to assess psychosocial functioning as a process clearly differentiated from psychopathological domains. This allows us to address the temporal paths between psychopathology and social functioning, which may provide information on the early developmental process of mental health problems in clinical and non-clinical settings, such as educational environments. Further research is required to shed light on individual differences between males and females in terms of psychosocial functioning in broader age ranges.

## Electronic supplementary material

Below is the link to the electronic supplementary material.

Supplementary Material 1

## Data Availability

The data that support the findings of this study are available from the corresponding author, [DN], upon reasonable request.
