# Supplementary material for: Initial validation of the Multidimensional Adolescent Functioning Scale (MAFS) in Spanish-speaking students from Chilean secondary schools
Source: Health Qual Life Outcomes. 2023 Jul 24;21:78. doi: 10.1186/s12955-023-02163-5 (PMC10367328; doi:10.1186/s12955-023-02163-5)
Supplement: Supplementary file 1 — Supplementary Material 1 [file 12955_2023_2163_MOESM1_ESM.docx]

**Supplementary tables**

**Table S1***Mean comparison for ERQ-CA, CBTSQ, SIQ-JR and PHQ-9 scores between male and female participants*

|  |  | Descriptives | | | | |  |  |  |  |
| --- | --- | --- | --- | --- | --- | --- | --- | --- | --- | --- |
|  |  | Male | |  | Female | |  | t-test | | |
|  |  | M | SD |  | M | SD |  | *t* | *p* | *d* |
| ERQ-CA | CR | 21.265 | 4.244 |  | 20.095 | 3.949 |  | 3.553 | .000 | .286 |
|  | ES | 12.851 | 3.499 |  | 13.681 | 3.194 |  | 3.087 | .002 | .248 |
| CBTSQ | CgR | 19.887 | 6.276 |  | 20.063 | 5.659 |  | .366 | .714 | .029 |
|  | BA | 20.844 | 6.071 |  | 18.145 | 5.827 |  | 5.644 | .000 | .454 |
| SIQ-JR | AI | 8.434 | 5.155 |  | 10.662 | 7.104 |  | 4.449 | .000 | .358 |
|  | GI | 13.987 | 7.967 |  | 17.432 | 9.697 |  | 4.817 | .000 | .387 |
|  | IP | 5.132 | 3.878 |  | 7.606 | 5.322 |  | 6.581 | .000 | .529 |
| PHQ-9 |  | 9.232 | 5.815 |  | 12.637 | 6.590 |  | 6.804 | .000 | .547 |

ERQ-CA=Emotion Regulation Questionnaire for Children and Adolescents; CR=Cognitive reappraisal; ES=Expressive suppression; CBTSQ=Cognitive Behavioral Therapy Skills Questionnaire; CgRs=Cognitive restructuring; BA=Behavioral activation; SIQ-JR=Suicide Ideation Questionnaire Junior; AI=Active ideation; GI=General ideation; IP=Interpersonal problems; PHQ-9=Patient health questionnaire; *t*=Student’s t statistic; *d*=Standardized mean difference

**Table S2***Correlation matrix for all measures*

|  |  | GF | FF | PF | CR | ES | AI | GI | IP | CgR | BA | PHQ-9 |
| --- | --- | --- | --- | --- | --- | --- | --- | --- | --- | --- | --- | --- |
| MAFS | GF | — |  |  |  |  |  |  |  |  |  |  |
|  | FF | .554** | — |  |  |  |  |  |  |  |  |  |
|  | PF | .328** | .291** | — |  |  |  |  |  |  |  |  |
| ERQ-CA | CR | .345** | .191** | .205** | — |  |  |  |  |  |  |  |
|  | ES | -.297** | -.248** | -.132** | .036 | — |  |  |  |  |  |  |
| SIQ-JR | AI | -.455** | -.317** | -.064 | -.253** | .258** | — |  |  |  |  |  |
|  | GI | -.525** | -.367** | -.107** | -.249** | .326** | .788** | — |  |  |  |  |
|  | IP | -.522** | -.405** | -.152** | -.267** | .037** | .759** | .805** | — |  |  |  |
| CBTSQ | BA | .562** | .372** | .463** | .377** | -.254** | -.235** | -.283** | -.308** | — |  |  |
|  | CgR | .080* | .033 | .174** | .148** | .012 | .134** | .216** | .085* | .398** | — |  |
| PHQ-9 | PHQ-9 | -.641** | -.399** | -.186** | -.287** | .397** | .609** | .720** | .662** | -.360** | .198** | — |

MAFS= Multidimensional Adolescent Functioning Scale; GF= General functioning; FF=Family functioning; PF=Peer functioning; ERQ-CA=Emotion Regulation Questionnaire for Children and Adolescents; CR=Cognitive reappraisal; ES=Expressive suppression; CBTSQ=Cognitive Behavioral Therapy Skills Questionnaire; CgRs=Cognitive restructuring; BA=Behavioral activation; SIQ-JR=Suicide Ideation Questionnaire Junior; AI=Active ideation; GI=General ideation; IP=Interpersonal problems; PHQ-9=Patient health questionnaire; *=*p*<.05; **=*p*<.001
